# Affordance Types and Their Determinants in Patient Mobile Health Portals Use: An Empirical Investigation

**DOI:** 10.2196/74351

**Published:** 2025-08-27

**Authors:** Haoran Zheng, Weidong Xia, Jingdong Ma

**Affiliations:** 1 Department of Management Information Systems, Foster College of Business, Bradley University, Peoria, IL, United States; 2 Department of Information Systems and Business Analytics, College of Business, Florida International University, Miami, FL, United States; 3 School of Medicine and Health Management, Tongji Medical College, Huazhong University of Science and Technology, No 13 Hangkong Road, Qiaokou District, Wuhan, 430030, China, 86 27 83692826

**Keywords:** hospital mobile health portals, general affordance, contextual affordance, behavioral affordance, patient absorptive capacity, mobile health portal innovativeness

## Abstract

**Background:**

Mobile health portals (MHPs) are becoming increasingly important and prevalent among health care organizations for engaging and retaining patients. However, their success hinges on patient adoption and usage. Organizations face ongoing challenges with high adoption but low usage of MHPs. While affordance theory offers a valuable theoretical perspective for exploring patient perceptions and uses of MHPs, there is a gap in research that empirically investigates the distinct types of affordances and their determinants.

**Objective:**

This study develops and empirically evaluates an integrated sociotechnical research model and hypotheses to bridge the literature gap by connecting theoretical constructs with empirical evidence and practical guidelines, thereby enhancing the understanding of patient MHP use. We extend the existing literature by differentiating 3 MHP affordance types: general, contextual, and behavioral. We examined the effects of patient absorptive capacity and hospital MHP innovativeness on the 3 types of affordances and their relationships. Based on the integrated sociotechnical research model and empirical results, we provide theoretical insights and practical guidelines on how organizations may enhance patient MHP use by enhancing patient absorptive capacity and improving patient-centered designs.

**Methods:**

We evaluated our research model and hypotheses using survey data from 401 patients regarding a WeChat-based MHP platform in 4 large urban hospitals in China. We pretested the measures via 3 rounds of Q-sorting, supplemented by field observations, expert reviews, and patient pilot tests. We tested the research model and hypotheses with partial least squares-structural equation modeling using the SmartPLS software.

**Results:**

General affordance was significantly affected by patient absorptive capacity (b=.191, *P*<.001) and MHP innovativeness (b=.567, *P*<.001). Contextual affordance was influenced significantly by patient absorptive capacity (b=.148, *P*<.001) and general affordance (b=.484, *P*<.001), but not by MHP innovativeness (b=.036, *P*=.564). Behavioral affordance was significantly influenced by contextual affordance (b=.430, *P*<.001) and general affordance (b=.140, *P*=.014). General affordance and contextual affordance mediate the effects of patient absorptive capacity and the innovativeness of hospital MHPs on behavioral affordance.

**Conclusions:**

Patient absorptive capacity and the innovativeness of MHPs in hospitals play distinct roles in predicting the 3 types of affordances. While the technology innovativeness of MHPs affects only general affordance but not contextual affordance, patient absorptive capacity affects both. Yet, contextual affordance is 3 times more effective in explaining behavioral affordance than general affordance. An unexpected finding related to the only unsupported hypothesis suggests that while the technology innovativeness of MHPs is necessary for general affordance, it does not directly influence MHP use; rather, it is indirectly and fully mediated by contextual affordance. Understanding and appropriately managing the contextual affordance of MHPs is critical in enhancing patient MHP use. To achieve the benefits of investing in MHPs, organizations must holistically understand and manage the 3 types of affordances, their determinants, and the relationships between them.

## Introduction

### Background

Health care organizations increasingly invest in patient-facing internet technologies such as mobile health portals (MHPs) to enhance patient engagement and experiences [1,2]. A recent study from HealthIT.gov reports that patient portal use has surged, with 75% of individuals nationwide being offered digital access to their medical records by health care providers or insurers in 2022, representing a 24% increase since 2020 [3]. While these health portals offer convenience and mobility to patients, their implementation and usage present significant challenges for health care organizations [4]. For example, although 75% of US adults were offered digital access to their medical records in 2022, 43% never logged in; among those who did, barely half used a smartphone app, and only one-third returned 6 or more times that year [3]. This usage gap highlights that technology alone does not guarantee patient engagement. Therefore, organizations have renewed emphasis on patient-centered care and elevating patient experience in the digital age [5]. Low adoption, underused features, and poor user experience often result in poor outcomes and reduce the return on investment for health care organizations [5-8].

The context of this research involves a mobile social media–enabled MHP-embedded application within China’s widely used social media platform, WeChat [9]. Most public hospitals embed MHP inside WeChat service accounts, riding on an ecosystem where mobile wallets handle most everyday transactions. Patients can use the MHP to schedule doctor appointments, check in at the hospital, make payments, access medical records, view laboratory results, refill prescriptions, consult with doctors digitally, and engage with other patients in web-based communities [7]. However, contextual constraints surrounding patients, such as digital literacy, health care infrastructures, regulations, and policies, often limit what patients can do with MHPs in a specific moment and context of use. These frictions may help explain why the MHP registration rate is generally high, yet the active use rate is low.

Early information systems (IS) research focused on technology features and user traits, while more recent studies use the concept of affordance to capture how material properties and social context interact during system use [10-12]. While affordance studies have informed system design [13], explained technology-driven organizational change [14,15], and explored users’ perceptions of functionality [2,16-18], a literature gap remains: we lack understanding of the different affordance types, their connections, or the social and technical factors that influence them.

To address these gaps, this study proposes an integrated model that expands the conceptualization of affordance by categorizing it into general, contextual, and behavioral affordances and incorporates both patient traits and MHP innovativeness to reflect the sociotechnical nature of MHP use. We collected survey data from 401 patients across 4 large hospitals in China to examine the effects of patient absorptive capacity and hospital MHP innovativeness on the 3 types of affordances and the relationships among these affordances. We developed and tested a model that examines the social and technical determinants of these 3 affordances. On the social side, we examine patients’ ability to understand and accept new applications like MHPs; on the technical side, we consider the innovativeness of the MHPs [19]. We empirically test our research model in the context of patient use of hospital MHPs. This paper aims to answer the following research question: what are the relationships among social and technical determinants and the 3 types of affordances in the context of patient use of hospital MHPs?

Our results reveal that contextual and general affordances are significant predictors of behavioral affordance, with contextual affordance being the stronger predictor. This confirms the crucial role that context plays in the use of MHPs. The findings also suggest a partial mediation effect of general affordance on behavioral affordance. Absorptive capacity has a positive impact on both contextual and general affordance. Interestingly, technology innovativeness positively predicts general affordance but not contextual affordance.

This study makes 2 key contributions. First, we enhance the existing conceptualization of affordance by categorizing it into 3 types: general, contextual, and behavioral affordances. The paper builds on the relational nature of affordance and captures the contextuality of sociotechnical interactions within daily operations in health care organizations [2,5,11,20]. Second, we provide insights into the relationships between the social and technical determinants and the 3 types of affordances. More specifically, we empirically test how patient absorptive capacity and perceived technology innovativeness influence patients’ general affordance, contextual affordance, and behavioral affordance in the context of hospital MHP [21]. The study results offer actionable guidelines for practitioners involved in the development, integration, and assimilation processes of social media-enabled health management systems [2].

### Theoretical Background

#### Concept of Affordance

The concept of affordance originates from Gibson [22], defined as the possibilities and limits for action that a material object offers to an actor. By focusing on the dynamic and contextual nature of user-technology interaction, researchers have defined IT affordance as a specific form of user-technology interaction. This notion arises from the traditional dichotomy between technological and social determinism, providing a basis for studying the co-evolution of humans and technology [12]. Affordance, as an intermediate approach to understanding technology, both connects and distinguishes itself from Giddens’ structuration theory [23], DeSanctis and Poole’s adaptive structuration theory [24], and Orlikowski and Scott’s social materiality [10]. While these approaches aim to explore the interplay among technology, actors, and organizations, they emphasize different aspects. Although the role of affordance in analyzing sociotechnical phenomena remains debated, its integration with critical theories further refines its perspective for studying sociotechnical interactions. Affordance, rooted in relational ontology, assigns equal importance to both the social and the material [25].

#### Types of Affordances

The IS literature has primarily recognized 2 distinct types of affordances. The first type, objective affordance, initially proposed by Gibson [22], refers to action possibilities that are inherently embedded in technological artifacts, independent of an actor’s experiences or perceptions. The second type, subjective affordance, or perceived affordance, emphasizes the user’s interpretation and recognition of these possibilities based on their knowledge and expectations, as articulated by Norman [26]. Affordances do not exist in isolation; they emerge dynamically and relationally through interactions among users, technologies, and contexts [27-30]. Yet, current research often neglects the complexities introduced by context, frequently treating affordances as stable and universally perceived technology features, without fully accounting for how contextual factors influence actual perceptions and usage behaviors [13,31].

We define and categorize affordances into 3 interconnected types: general, contextual, and behavioral. Table 1 summarizes the proposed types of affordances. General affordance refers to users’ stable, baseline perceptions about the functionalities and capabilities of an artifact, shaped by their prior knowledge and long-term expectations. Contrarily, contextual affordance represents dynamic, immediate perceptions formed in the moment of interaction between the user, the technology, and the specific circumstances, influenced by situational variables such as current needs, urgency, environment, or institutional constraints. Behavioral affordance captures the actual, enacted behaviors resulting from users’ perceptions, representing successful MHP usage.

**Table 1.** Three affordance types.

Affordance type	Characteristic	Description	Example
General	Baseline: long-term, distal, continuous, rigorous, and stable	What patients believe the portal can do, based on prior knowledge	A patient believes what she or he can use an MHP^a^ to book a doctor’s appointment
Contextual	Dynamic: short-term, immediate, situational, and unstable	What the portal allows the patient to do in this specific situation	A patient can book a doctor’s appointment using an MHP up to 7 days in advance
Behavioral	Actual use: enacted and realized	What patients’ actual use of MHPs’ features entails	A patient uses an MHP to book a doctor’s appointment

a MHP: mobile health portal.

When applying affordance theory, scholars often frame affordance as interactions among actors, artifacts, or processes, yet frequently overlook the specific context in which these interactions unfold. For instance, Volkoff and Strong [31] argue that affordance represents a specific instance of generative mechanisms. They investigate technology-induced generative mechanisms that interact with affordances during an IT-enabled change process. However, they overlook non–technology-related generative mechanisms that may also interact with affordances. Although the importance of context in affordance theory is well recognized, there remains a lack of clear conceptualization for context-sensitive affordances. Markus and Silver [13] note that no explanation of IT effects would be complete without careful conceptualizations of users and their use environments. These environments include users’ characteristics, goals, interpretations of technology, work practices, and institutional contexts.

An MHP should be seen as a social object, not merely a functional tool. These 3 affordance types are closely related and interact with each other. Contextual affordance temporarily refines or modifies users’ general affordance during specific interactions, guiding the emergence of behavioral affordance. Thus, the interplay among these 3 affordance types highlights how stable expectations and immediate contextual conditions together shape the actions users ultimately perform. Understanding the nuanced relationships among general, contextual, and behavioral affordances can provide deeper insights into both theory development and the practical design of IS, thereby enhancing MHP use and overall user experience. Affordance connects technology and actors and is inseparable from social characteristics [14,32,33]. Thus, our integrated framework includes both technology innovativeness and patients’ absorptive capacity.

#### Technology Innovativeness

Technology innovativeness is defined based on the diffusion of innovation theory [34-36], which seeks to explain how, why, and at what rate innovations and technologies spread. The theory has been used to assess behaviors related to the adoption of new technological innovations. Diffusion is defined as a process by which an innovation is communicated through specific channels among members of a social system over time. In total, 5 characteristics of innovation influence its adoption: relative advantage, compatibility, complexity, trialability, and observability [34]. In addition to these 5 characteristics, prior conditions such as previous practice, needs, innovativeness, and social system norms are also considered in adoption research [37]. In this study, technology innovativeness reflects users’ perceptions of the MHP’s novelty and usefulness.

#### Absorptive Capacity

Absorptive capacity refers to one’s ability to identify, assimilate, and exploit knowledge from the environment [38]. It encompasses the recipients’ capacity to absorb and make use of transferred knowledge during this process. Absorptive capacity shapes implementation outcomes by enabling recipients to assimilate and apply knowledge [39]. In our research, absorptive capacity maintains its original definition and illustrates the degree to which a patient can identify, assimilate, and exploit the knowledge of the hospital MHP.

### Research Model and Hypotheses

**Figure 1.**
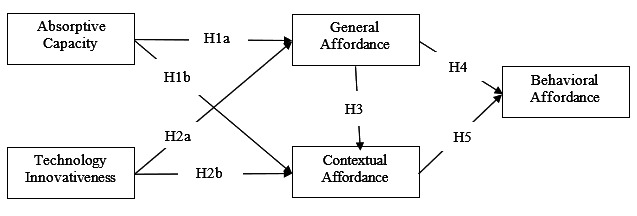
Research model.

Building on the above theoretical foundation, we propose an integrated research model and hypotheses that expand affordance theory by categorizing affordance into general, contextual, and behavioral affordances and account for the sociotechnical nature of MHP through both patient traits and perceived MHP innovativeness. Figure 1 illustrates the overview of our research model.

### Absorptive Capacity and General Affordance

Absorptive capacity refers to an individual’s ability to recognize, assimilate, and apply new information effectively [38]. IS implementation involves knowledge transfer, where successful adoption and use depend heavily on the user’s capability to absorb this knowledge [40]. Patients with higher absorptive capacity are more likely to form accurate expectations about what the MHP generally enables, even before direct interaction [39]. Thus, patients’ absorptive capacity positively influences their baseline perceptions: the general affordance of the functionalities the MHP provides. We propose that patients’ absorptive capacity is positively related to general affordance (hypothesis 1a).

### Absorptive Capacity and Contextual Affordance

Contextual affordance refers to the patient’s evaluation of action possibilities with the MHP at a specific moment, considering immediate contextual factors such as payment methods, available functionalities, and access constraints [41]. Previous studies suggest that absorptive capacity significantly impacts the use of technology and the contextual understanding of available system features [42,43]. Patients with high absorptive capacity better adjust their perceptions from general possibilities to context-specific realities, effectively identifying viable options during MHP use. We propose that patients’ absorptive capacity positively relates to patient-perceived contextual affordance (hypothesis 1b).

### Technology Innovativeness and General Affordance

According to diffusion of innovation theory, technology innovativeness significantly shapes user perceptions about what the characteristics of the technology mean to them, including compatibility, relative advantage, and usability [34,37,44]. Patients assess MHPs’ innovativeness based on how well it fits their existing ways of doing things and anticipated practical benefits [45]. These perceived innovative features positively influence patients’ general expectations and assumptions about the portal’s capabilities. Therefore, we propose that patient-perceived technology innovativeness of MHPs is positively related to patient general affordance (hypothesis 2a).

### Technology Innovativeness and Contextual Affordance

When patients use MHPs in a specific context, they quickly form an opinion on how to leverage it to their advantage and which functionalities to use. With these goals, patients can move forward with their interactions. The concept of contextual affordance differs from general affordance. In cases where patients engage with the MHPs’ functionalities, the evaluation of such a fit will be constrained to the actual functionalities of MHPs and other contextual factors, such as time, location, and internet access. We propose that patient-perceived technology innovativeness of MHPs is positively related to patient contextual affordance (hypothesis 2b).

### General and Contextual Affordances

General affordance represents stable, baseline perceptions derived from prior experiences, serving as a foundation for contextual affordance. Contextual affordance modifies these perceptions based on immediate environmental conditions and specific portal functionalities encountered in real time [20,46]. Patients’ general beliefs about MHPs’ capabilities influence how readily they perceive and accept specific functionalities during actual interactions. Thus, general affordance positively influences contextual affordance. We propose that patients’ general affordance is positively related to contextual affordance (hypothesis 3).

### General and Behavioral Affordances

In general, how patients perceive what MHPs can do for them influences how they use it [2]. Contextual affordance refers to the perceived action possibilities that the MHP affords to the patient when they need to interact with it in a specific use context. Therefore, contextual affordance contributes to the subsequent behavioral affordance (eg, what functionalities of the MHP the patient uses and how often they use them). In this study, behavioral affordance is defined as the actualized affordance, which denotes the actual use of the MHPs’ features. We propose that patients’ general affordance is positively related to behavioral affordance (hypothesis 4).

### Contextual and Behavioral Affordances

Contextual affordance involves immediate, situation-specific perceptions of MHPs’ functionality. Norman [26] emphasizes that subjective affordances are shaped by individual and cultural conventions, which influence user behavior. Previous research confirms that clearly perceived contextual possibilities strongly drive actual user actions [2]. Thus, when patients distinctly perceive actionable options at the point of interaction, they are more likely to translate these affordances into concrete behaviors. We propose that patients’ contextual affordance is positively related to behavioral affordance (hypothesis 5).

## Methods

### Data Collection

Our survey study was approved by the Social and Behavioral Institutional Review Board of the principal investigator’s university. The study sites consisted of 4 large urban hospitals in China, selected using a convenience sampling approach. At the time of data collection, all participating hospitals had implemented WeChat-based patient portals. Paper-based questionnaires were used to collect data on-site. After obtaining permission from each participating hospital, research team members approached patients onsite randomly to invite them to participate. Participants were not selected based on any specific characteristics; all eligible patients had an equal opportunity to be approached. Patients were eligible if they were adults, not sleeping, and able to understand and answer the survey questions. Before completing the survey, participants received an information sheet describing the study’s purpose, emphasizing that participation was entirely voluntary, responses would be analyzed only in aggregate for research purposes, and individual responses would remain confidential and anonymous in any resulting publications or reports. All respondents participated voluntarily. The main reasons patients did not agree to participate were that they were in a rush, did not use the patient portal, or were unable to understand and answer the survey questions. A total of 401 valid survey responses were collected and used in our analyses.

### Ethical Considerations

The Institutional Review Board of Florida International University granted the ethical approval (reference #: IRB170350) for this study.

### Measurement

Measurement items were adapted from the extant literature and further validated using our study sample. The measurement items used to assess each construct are listed in Multimedia Appendix 1. Absorptive capacity and technology innovativeness were modeled as reflective constructs, while contextual, general, and behavioral affordances were modeled as formative constructs. All measures were assessed using a 7-point Likert scale, ranging from 1 (strongly disagree) to 7 (strongly agree).

For absorptive capacity, 4 items from previous research [47-49] were adopted to capture a patient’s ability to recognize, assimilate, and apply new knowledge. The unit of analysis for previous instruments on absorptive capacity was primarily organizational; thus, we modified the items to an individual level in our study context. For technology innovativeness, we adapted items from widely accepted instruments [49] on perceptions of adopting an IT innovation based on the 5 innovation characteristics proposed by Rogers [36]. We modified these items to align with the context of our study.

We adapted measures based on previous research [1,2,11,31] for general, contextual, and behavioral affordance. General affordance was assessed using 4 items that capture patients’ perceptions, without referring to their specific medical conditions, regarding MHPs’ features that enable them to schedule appointments, make payments, interact and consult with doctors online, and access hospital information. Contextual affordance was evaluated with 4 items that gauge patients’ perceptions about their specific medical conditions, their ability to use the MHP to schedule appointments, make payments, access laboratory results and medical records, and ask medical staff questions online. Behavioral affordance was examined using 4 items that reflect the patients’ actual use of MHP to make appointments with their preferred doctors, make payments, access laboratory results, and consult with doctors online.

We conducted 3 rounds of Q-sorting to refine the initial measures. As a part of the pretest, we conducted field observations and interviews at the research sites. We pilot-tested the survey items with subject experts and patients to ensure their clarity and scope coverage for our study.

### Data Analysis

The study uses structural equation modeling (SEM) using partial least squares (PLS) analysis with the SmartPLS software. We first assessed the reliability and construct validity of the measurement. For reflective variables, construct reliability was measured with Cronbach α, composite reliability, rho_a, and rho_b, with acceptance criteria of 0.7 or above for all 4 [50,51]. Convergent validity was evaluated by examining the average variance extracted, with an acceptance criterion of 0.5 or above [52,53]. Discriminant validity was assessed using the Fornell-Larcker criterion (square root of the average correlations’ variance greater than the correlations between the latent variables), cross-loadings (above 0.5), and the heterotrait-monotrait ratio (below 0.85) [52,54,55].

For formative constructs, variance inflation factor (VIF), weights, and item significance were tested. Multicollinearity is a key concern for formative indicators [56], as the model relies on multiple regression. VIF value should exceed 0.20 and be below the common threshold of 10 [56]. Outer weights assess the contribution of each indicator to its construct, using latent variable scores as dependent variables and formative indicators as predictors in a multiple regression analysis. The values of outer weight express each indicator’s relative contribution to the construct, with an acceptance criterion of 0.5 or above [51].

After validating the measurement model, we assessed our structural model to test our hypotheses. Lastly, we conducted additional analyses to examine the mediating effects embedded in our research model [57]

Ensuring an adequate sample size is critical in PLS-SEM. A common rule of thumb requires at least 10 observations per indicator or per structural path in the most complex model segment [52,54]. Monte Carlo simulations also recommend a minimum of 200 cases to ensure stable parameter recovery [58]. Our study surpasses all these thresholds, providing sufficient statistical power, reliable path estimates, and precise confidence intervals for robust PLS-SEM analysis. As shown in Table 2, our sample was about evenly split by gender. Educational attainment spanned from primary school through doctoral degrees, 1 in 3 held a bachelor’s degree, and just 1.0% had earned a doctorate. Employment profiles showed private-sector work accounted for nearly half the sample.

**Table 2.** Sample characteristics.

Characteristics	Participants, n (%)
Gender, n (%)	
Male	182 (45.4)
Female	211 (52.6)
Missing	8 (2)
Age group (years), n (%)	
<18	5 (1.2)
18‐30	184 (45.9)
31‐40	86 (21.5)
41‐50	45 (11.2)
>50	57 (14.2)
Missing	24 (6)
Education level, n (%)	
Doctoral	4 (1)
Graduate	30 (7.5)
Bachelor	139 (34.7)
Technical college	65 (16.2)
Vocational	22 (5.5)
Senior high	62 (15.5)
Junior high	33 (8.2)
Primary school	34 (8.5)
Missing	12 (2.9)
Purpose for visit, n (%)	
First timer	118 (29.4)
Follow-up	113 (28.2)
Accompany	139 (34.7)
Not specified	18 (4.5)
Missing	13 (3.2)
Employer type, n (%)	
Government	12 (3)
State-owned	33 (8.2)
Private-owned	123 (30.7)
Foreign-owned	67 (16.7)
Civil enterprises	23 (5.7)
Small business	66 (16.5)
Farmer	32 (8)
Others	30 (7.5)
Missing	15 (3.7)

## Results

### Reliability and Validity for Constructs

The reliability and validity test results of the constructs are presented in Table 3. For the two reflective constructs, patient absorptive capacity and technology innovativeness, Cronbach α values were satisfactory. In addition to Cronbach α, rho_a, rho_c, and composite reliability for the two reflective constructs indicated satisfactory reliability. For convergent validity, all indicators of our two reflective constructs met these benchmarks. Results demonstrate that the average variance extracted for reflective constructs was 0.86 and 0.87, and all outer loadings were 0.85 or greater. These findings indicate that the measures used for absorptive capacity and technology innovativeness constructs showed adequate convergent validity. Discriminant validity is confirmed when diagonal elements exceed the off-diagonal values in the corresponding rows and columns. Our results demonstrate that the two reflective constructs exhibit sufficient discriminant validity. Therefore, the two reflective constructs, patient absorptive capacity and technology innovativeness, demonstrated adequate reliability and construct validity.

For our 3 formative constructs, general affordance, contextual affordance, and behavioral affordance, as shown in Table 3, each indicator’s VIF value was significantly below the standard cutoff threshold score of 10 [56]. Therefore, multicollinearity was not a concern in our formative constructs. We observed small to moderate weights for most items, from 0.11 to 0.61. We used bootstrapping to test whether the formative measurement models’ outer weights differed significantly from zero. Bootstrapping results showed significant *P* values for most formative indicators. In total, 3 items in behavioral affordance were insignificant, with *P* values at 0.07, 0.08, and 0.33; 1 item in contextual affordance (*P*=.37) was insignificant. These items were retained due to their unique theoretical contributions to construct validity.

**Table 3.** Reliability and validity tests for the constructs.

Item	Loadings/weights	VIF^a^	*P* value	AVE^b^	Cronbach α	rho_a	rho_c
Reflective constructs							
Absorptive capacity (AC)		—^c^	—	0.744	0.889	0.939	0.921
AC1	0.857						
AC2	0.865						
AC3	0.871						
AC4	0.857						
Tech innovativeness (TI)		—	—	0.748	0.916	0.918	0.937
TI 1	0.847						
TI 2	0.859						
TI 3	0.870						
TI 4	0.884						
TI 5	0.864						
Formative constructs							
General affordance (GA)				—	—	—	—
GA1	0.467	2.086	.001				
GA2	0.308	2.468	.001				
GA3	0.236	2.018	.001				
GA4	0.179	1.530	.01				
Contextual affordance (CA)				—	—	—	—
CA1	−0.096	3.059	.44				
CA2	0.215	1.406	.007				
CA3	0.399	2.008	.001				
CA4	0.644	2.828	.001				
Behavioral affordance (BA)				—	—	—	—
BA1	0.425	2.526	.003				
BA2	0.343	3.613	.06				
BA3	0.170	3.449	.32				
BA4	0.199	3.449	.10				

a VIF: variance inflation factor.

b AVE: average variance extracted.

c Not applicable.

### Structural Model Testing

### Hypothesis Testing

**Figure 2.**
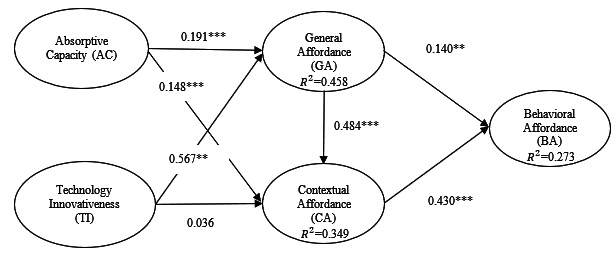
Structural equation estimate results.

The next step in our data analysis was to evaluate the structural model by examining the path coefficient (*β*) and variance explained (*R*^2^) [59]. *R*^2^ represents the amount of variance in the construct in question that the model explains. The test results of the path model are presented in Figure 2 and Table 4. An *R*^2^ value of 0.273 indicates that 27.3% of the variance in behavioral affordance was explained by contextual and general affordance, absorptive capacity, and technology innovativeness.

**Table 4.** Structural model validation and hypothesis testing results.

Path	Original sample	SD	*t* test (*df*)	*P* values	Hypothesis testing
H1a^a^: AC→GA	0.191	0.047	4.116 (400)	.001	Supported
H1b^b^: AC→CA	0.148	0.053	2.812 (400)	.005	Supported
H2a^c^: TI→GA	0.567	0.047	11.971 (400)	.001	Supported
H2b^d^: TI→CA	0.036	0.062	0.578 (400)	.56	Not supported
H3^e^: GA→CA	0.484	0.064	7.527 (400)	.001	Supported
H4^f^: GA→BA	0.140	0.057	2.471 (400)	.01	Supported
H5^g^: CA→BA	0.430	0.059	7.312 (400)	.001	Supported

a H1a: patients’ absorptive capacity positively affects general affordance.

b H1b: patients’ absorptive capacity positively affects contextual affordance.

c H2a: perceived technology innovativeness for patients positively affects affordance.

d H2b: perceived technology innovativeness for patients positively affects contextual affordance.

e H3: general affordance has a positive effect on contextual affordance.

f H4: general affordance has a positive effect on behavioral affordance.

g H5: contextual affordance has a positive effect on behavioral affordance.

Results show that patients’ absorptive capacity positively influenced both general and contextual affordances. Perceived technology innovativeness was positively related to general affordance but not contextual affordance. General affordance has a positive effect on both contextual and behavioral affordance. Lastly, contextual affordance was positively associated with behavioral affordance. Therefore, hypotheses 1a, 1b, 2a, 3, 4, and 5 were supported, whereas hypothesis 2b was not. The effects of technology innovativeness on general affordance, general affordance on contextual affordance, and contextual affordance on behavioral affordance were larger than those of the other paths.

These results indicate that increased absorptive capability, which captures a patient’s ability to recognize, assimilate, and apply new knowledge toward MHP, contributes to a higher level of perceived affordance, regardless of the type of affordance. Furthermore, our result related to hypothesis 2b, which was not supported, was particularly interesting. One explanation is that while MHP technology’s innovativeness, defined by designed features, may shape patients’ static perception of what general actions those features can enable them to perform, it does not address the idiosyncratic needs of patients in specific use contexts. As such, MHP technology’s innovativeness influences general affordance directly but not contextual affordance. Contextual affordance (*β*=.430) had a 3-fold stronger effect on behavioral affordance compared to general affordance (*β*=.140).

### Mediating Analyses

Mediation analysis provides insights into the mechanisms underlying MHP usage through the lens of affordance [60]. This information further enriches our comprehension of the effects of affordance, potentially guiding the discovery of more effective and cost-efficient strategies to enhance patient portal use. The details are presented in Table 5.

**Table 5.** Direct, total indirect, and specific indirect effects.

	Original sample	Sample mean	SD	*t* test (*df*)	*P* value
Direct effects					
AC^a^→BA^e^	0.246	0.247	0.057	4.333 (400)	.001
TI^b^→BA	−0.003	−0.001	0.061	0.052 (400)	.96
Total indirect effects					
AC→BA	0.103	0.104	0.027	3.786 (400)	.001
TI→BA	0.152	0.152	0.036	4.204 (400)	.001
Specific indirect effects					
AC→GA^c^→BA	0.011	0.010	0.012	0.878 (400)	.38
AC→GA→CA^d^	0.092	0.093	0.025	3.640 (400)	.001
AC→GA→CA→BA	0.035	0.036	0.012	3.043 (400)	.002
AC→CA→BA	0.057	0.058	0.022	2.593 (400)	.01
TI→GA→CA	0.275	0.278	0.044	6.327 (400)	.001
TI→GA→BA	0.032	0.029	0.035	0.902 (400)	.37
TI→GA→CA→BA	0.106	0.109	0.023	4.690 (400)	.001
TI→CA→BA	0.014	0.014	0.025	0.551 (400)	.58

a AC: absorptive capacity.

b TI: technological innovativeness.

c GA: general affordance.

d CA: contextual affordance.

e BA: behavioral affordance.

Absorptive capacity had a significant direct effect on behavioral affordance (*β*=.246, *t_400_*=4.333, *P*<.001), whereas technology innovativeness did not (*β*=−.003, *t*_400_=0.052, *P*=.959). Both contextual (*β*=.430, *t*_400_=7.312, *P*<.001) and general affordance (*β*=.140, *t*_400_=2.471, *P*=.014) positively influenced behavioral affordance.

Absorptive capacity had a significant indirect effect on behavioral affordance (*β*=.103, *t*_400_=3.786, *P*<.001), indicating partial mediation through affordances. In contrast, technology innovativeness did not directly affect contextual affordance but instead did so indirectly through general affordance. This suggests that general affordance fully mediated the relationship between technology innovativeness and contextual affordance. Technology innovativeness also had a significant indirect effect on behavioral affordance (*β*=.152, *t*_400_=4.204, *P*<.001), but since the direct effect was not significant, general and contextual affordances fully mediated this relationship.

These results highlight that general and contextual affordances are crucial in translating absorptive capacity and technology innovativeness into behavioral affordance. While absorptive capacity directly influences behavioral affordance through general and contextual affordances, technology innovativeness impacts behavioral affordance only through general and contextual affordances. This underscores the importance of designing MHPs that enhance affordance perception to facilitate patient engagement and use.

## Discussion

### Summary of Results

This study develops and tests an integrated sociotechnical model that considers the interrelationships among patient absorptive capacity and 3 types of affordances: general, contextual, and behavioral. The model was tested using PLS-SEM with a sample of 401 patients from 4 large urban hospitals in China. Of the 7 hypothesized relationships, 6 were supported and one was not. Patient absorptive capacity significantly affected both general and contextual affordances. MHP innovativeness significantly affected general affordance but not contextual affordance. General affordance affected behavioral affordance both directly and indirectly through contextual affordance. Contextual affordance had a much stronger effect on behavioral affordance than general affordance. While MHP innovativeness did not directly affect contextual affordance, it indirectly affected contextual affordance through general affordance. In addition, general affordance and contextual affordance mediate the effects of patient absorptive capacity and the innovativeness of hospital MHP on behavioral affordance.

### Theoretical Contributions

This study makes 3 theoretical contributions to the literature. First, we distinguish 3 affordance constructs in the context of health care research: general, contextual, and behavioral affordance. General affordance is conceptualized as the patients’ evaluation of what they can do with the MHP before specific use in a given context. The perceived possible actions afforded to the patients by MHP largely depend on the patients’ prior experience with similar technology. In contrast, contextual affordance refers to the patient’s evaluation of what they can do with the MHP at the point of interaction, which is inherently embedded in that context. Contextual affordance and general affordance are distinct and affect patient behavior differently. Contextual affordance is more immediate, unstable, and variable. It comes into play when a patient must decide whether to use the patient portal. When contextual affordance is present, the more stable, baseline-level general affordance is temporarily adjusted. We shift the focus from the primary treatment of affordance from system features to examining contextualized action possibilities, accounting for both the baseline affordance and affordance in action. This notion differs from previous empirical research on affordance [61,62].

Second, we empirically investigated how contextual affordance relates to, but differs from, general affordance in influencing behavioral affordance. This study responds to calls for research that examines different contexts when analyzing the relationship between technology (the artifact) and actors (the social) [10,63]. Using a sociotechnical perspective, we address the need to use the lens of affordance to examine its actualization in health care contexts [64]. Strong and Volkoff [64] introduced the concept of a bundle of interrelated and interacting affordances. Consistent with this concept, rather than assuming that affordance is a singular, stable construct, we further differentiate contextual affordance from general affordance. We tested these effects across 4 large hospitals in China.

Third, we examine 2 critical determinants of affordances, whereas prior research generally treats affordances as independent variables [61,65]. The contingency hypotheses for absorptive capacity and technology innovativeness capture how patients form affordances for the MHP. Consistent with previous research [39,45], absorptive capacity captures the social aspect of MHP through users’ capability to evaluate MHP, whereas technology innovativeness captures the technical elements of MHP [63]. Both play key roles in forming patients’ affordance. While Hypothesis H2b was not supported, the result represented an interesting finding. The lack of a direct effect of MHP technology innovativeness on contextual affordance can be explained by our mediation analysis, which showed that the effect of technology innovativeness on contextual affordance was fully mediated by general affordance. These results imply that MHP technology innovativeness, as predetermined by the designed technical features, directly influences patients’ static perceptions of what those features enable them to do. Technology innovativeness’s lack of direct effect on contextual affordance suggests that the predetermined technical features, while important in influencing patients’ general affordance, cannot capture the emerging needs of patients in their idiosyncratic use of MHP. Given the practical challenges that organizations face with high adoption but low use of MHP [3], based on our findings, we speculate that while general affordance explains patient adoption, contextual affordance is a better indicator explaining patient usage. Our results are consistent with Strong and Volkoff [64], who contended that other factors, such as users’ goals and immediate outcomes, will affect the actualization of affordance.

### Practical Implications

MHP serves as a powerful tool for organizations to gain a competitive advantage and adapt to ever-changing patient demands [66]. While MHP investments benefit some organizations, results for others are mixed due to insufficient consideration of context when adopting, implementing, and disseminating these technologies [10]. Through the lens of affordance, we urge organizations to incorporate contextual elements into their planning and evaluation of technology investments and usage [63]. Health care organizations can better convert MHP investments into enhanced patient use when contextual aspects are thoroughly considered [5,63,67]. Our results support the hypothesis that the positive effects of affordance influence behavioral affordance through MHP use.

The concept of behavioral affordance aligns with prior research advocating for a nuanced distinction between affordance and its actualization in a dynamic process [64,68]. Our results suggest that general affordance influences patient behavioral affordance through contextual affordance. This implies that patients’ decisions to use the MHP are contingent not only on the general affordances they possess before using it but also on the contextual affordances established during the interaction [63,67]. Furthermore, our findings reveal that contextual affordance has a more significant impact on behavioral affordance than general affordance. Patients’ decisions to use the MHP are firmly rooted in contextual affordance at the specific moment of interaction rather than in the level of general affordance prior to those interactions [5]. A 1 SD lift in contextual affordance yields more behavioral affordance gain comparable to adding all other predictors in the model combined. At the same time, general affordance fully mediated the relationship between MHP technology innovativeness and contextual affordance.

For designers and managers, our findings suggest that while general affordance, in and of itself, does not directly affect contextual affordance, it serves as a necessary mediator. Managers and designers should carefully consider these relationships when planning and implementing the MHP technical features. Core features addressing stable patient needs should be well-designed, while dynamic and context-sensitive features should also be prioritized. For example, dynamic features such as showing live queue length immediately before booking or surfacing a one-tap payment link at the end of a patient visit or consultation may boost usage more than launching new, context-neutral features. Additionally, we observe that technology innovativeness affects general affordance more than contextual affordance, while absorptive capacity has a stronger effect on contextual affordance than on general affordance.

### Limitations and Future Research

Due to the limitations of our study, the results should be interpreted with caution. While this research represents one of the early empirical examinations of the 3 types of affordances, limited generalizability and potential biases must be acknowledged. First, due to our convenient research site sampling of 4 large urban hospitals in China, the generalizability of our study findings must consider the limitations related to hospital size (large), geographic location (urban), and country (China). Future research should examine the generalizability of our study findings in other contexts that differ from ours in terms of hospital size, geographic location, and country.

Second, although we approached participants randomly onsite at the hospitals, we did not have a way to statistically examine selection biases on both our side and the patients’ side. Patients who did not participate were mainly those who did not have time, did not use the patient portals, or were unable to understand and answer the survey questions. Future research should consider statistically testing selection biases. In addition, we did not have a priori population lists of patients. As such, we could not use a formal randomization method to identify and recruit patients on an a priori basis. Future studies should use rigorous random sampling to ensure that the study sample is representative of the entire population and that there are no selection biases.

Based on our findings on the relationships between technology innovativeness, general affordance, and contextual affordance, we speculated that general affordance may predict patient MHP adoption. In contrast, contextual affordance may be a better predictor of patient MHP use. Consistent with Strong and Volkoff’s [64] call to consider contextual factors such as users’ goals and immediate outcomes in studying actualization of affordance, we encourage future research to examine the different roles of general affordance and contextual affordance in predicting patient MHP adoption and use.

### Conclusions

This study offers key insights into the factors influencing patient use of hospital MHP by proposing an integrated framework that links 3 types of affordances through a sociotechnical lens. Our research demonstrates that patient absorptive capacity and the innovativeness of hospital MHP are key predictors of these affordances. Specifically, absorptive capacity was found to positively influence both general and contextual affordances, which in turn positively affect behavioral affordance. The findings highlight the critical role that contextual factors play in system use, as contextual affordance emerged as a stronger predictor of behavioral affordance than general affordance. The study also reveals that technology innovativeness is a significant predictor of general affordance, but its influence on contextual affordance is not direct but rather indirect and fully mediated by general affordance. This suggests that the immediate context of system use plays a more crucial role in patient behavior than the perceived innovativeness of the technology itself. These findings underscore the importance of considering both the technical and social dimensions of affordances in the design and implementation of MHP. For practitioners, this research provides actionable guidelines for enhancing patient portal use by focusing on the management of contextual affordances at the point of interaction.

## Supplementary material

10.2196/74351 Multimedia Appendix 1 Measurement items.

## Abbreviations

IS: information system
MHP: mobile health portal
PLS: partial least squares
SEM: structural equation modeling
VIF: variance inflation factor
