# Supplementary figures and images for: Affordance Types and Their Determinants in Patient Mobile Health Portals Use: An Empirical Investigation
Source: J Med Internet Res. 2025 Aug 27;27:e74351. doi: 10.2196/74351 (PMC12387380; doi:10.2196/74351)

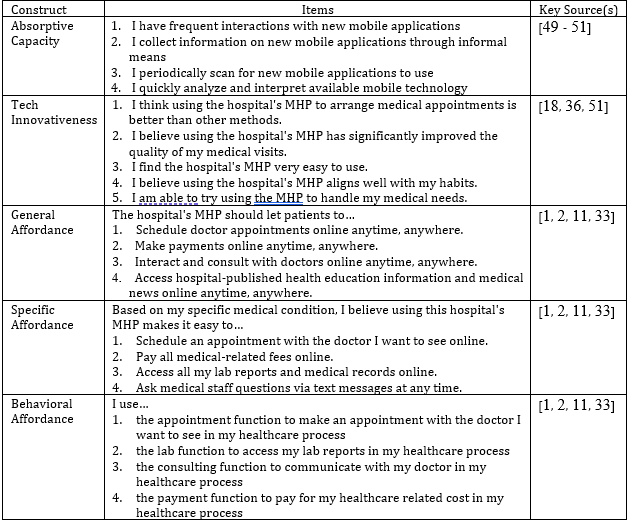

Supplement: Multimedia Appendix 1 [file jmir-v27-e74351-s001.png]
